# Supplementary material for: Development of a computer-aided design software for the quantitative evaluation of aesthetic damage
Source: PLoS One. 2019 Dec 18;14(12):e0226322. doi: 10.1371/journal.pone.0226322 (PMC6919621; doi:10.1371/journal.pone.0226322)
Supplement: S1 Code site — 1. (ZIP) [file pone.0226322.s001.zip › [EESC_jr][Projeto]Relatorio_07_21/vendors/select2/docs/_includes/options/selections/multiple.html]

## Can I allow users to make multiple selections?

Yes, Select2 supports making multiple selections through the use of the `multiple` option that can be passed in when initializing Select2.

### Can the `multiple` attribute be used on my `<select>` element?

Yes, Select2 will automatically map the value of the `multiple` attribute to the `multiple` option during initialization.
